# PD-1单抗导致免疫检查点抑制剂相关肺炎1例——吡非尼酮治疗的安全性和有效性

**DOI:** 10.3779/j.issn.1009-3419.2021.103.08

**Published:** 2021-07-20

**Authors:** 海明 于, 进英 李, 兰 于, 曦 程, 晓娜 韩, 小涛 张

**Affiliations:** 266042 青岛，青岛市中心医院立体定向放疗科 Department of Stereotactic Radiotherapy, Qingdao Central Hospital, Qingdao 266042, China

**Keywords:** 免疫检查点抑制剂, 肺炎, PD-1单抗, 吡非尼酮, 肺肿瘤, Immune checkpoint inhibitors, Pneumonia, PD-1 monoclonal antibody, Pirfenidone, Lung neoplasms

## Abstract

**背景与目的:**

免疫检查点抑制剂相关肺炎（checkpoint inhibitor pneumonitis, CIP）是严重的免疫检查点抑制剂副反应，急性期治疗手段已有共识，但急性期之后的肺间质纤维化治疗手段仍是临床需要解决的问题。

**方法:**

回顾性分析了青岛市中心医院立体定向放疗科收治的1例细胞程序性死亡受体1（programmed cell death1, PD-1）单抗导致免疫检查点抑制剂相关肺炎的非小细胞肺癌（non-small cell lung cancer, NSCLC）患者的诊断、治疗过程，并文献复习。

**结果:**

患者男性，70岁，初始诊断：左肺低分化鳞癌T3N3M0 Ⅲc期纵隔淋巴结转移表皮生长因子受体（epidermal growth factor receptor, EGFR）/间变性淋巴瘤激酶（anaplastic lymphoma kinase, ALK）/原癌基因1酪氨酸激酶（*C-ros* oncogene 1 receptor tyrosine kinase, *ROS1*）/RAF阴性PD-L1（22C3）阴性。一线化疗进展后纳武利尤单抗单药二线治疗过程中确诊为免疫检查点抑制剂相关肺炎3级。初始大剂量糖皮质激素冲击治疗后患者肺部计算机断层扫描（computed tomography, CT）影像学和临床症状部分缓解，随后给予吡非尼酮口服（300 mg *tid*）11个月余，治疗过程中患者CT影像学和临床症状明显好转，除1级恶心外无其他不良反应。期间吡非尼酮与化疗、安罗替尼联合应用安全性好。

**结论:**

本病例报道为目前吡非尼酮治疗CIP的第1例报道，为CIP治疗的临床实践和临床研究提出了新的思路。

## Abstract (en)

**Background and objective:**

Immune checkpoint inhibitor associated pneumonia (CIP) is a serious side effect of immune checkpoint inhibitors. There is a consensus on the treatment of acute phase of CIP, but the treatment of pulmonary interstitial fibrosis after the acute phase is still a clinical problem to be solved.

**Methods:**

The diagnosis and treatment of a non-small cell lung cancer (NSCLC) patient with immune checkpoint inhibitor associated pneumonia in the Stereotactic Radiotherapy Department of Qingdao Central Hospital were retrospectively analyzed, and literatures were reviewed.

**Results:**

A 70-year-old male patient was diagnosed with Poorly differentiated squamous cell carcinoma of left lung with mediastinal lymph node metastasis T3N3M0 stage Ⅲc, EGFR/ALK/ROS1/RAF negative, PD-L1 (22c3) immunohistochemistry negative. After the progression of first-line chemotherapy, the patient was diagnosed as immune checkpoint inhibitor associated pneumonia grade 3 during second-line monotherapy with Nivolumab. After initial high-dose glucocorticoid pulse therapy, the lung computed tomography (CT) imaging and clinical symptoms of the patients were partially relieved, and then pirfenidone (300 mg *tid*) was given orally for more than 11 months. During the treatment of pirfenidone, the CT imaging and clinical symptoms of the patients were significantly improved, and there were no other adverse reactions except grade 1 nausea. During this period, chemotherapy and Anlotinib was given concurrently with pirfenidone and showed good safety profile.

**Conclusion:**

This case report is the first report of pirfenidone in the treatment of CIP, which provides a new idea for the clinical practice and research of CIP treatment.

## 1 病例资料

### 1.1 一般资料及确诊

患者男性，70岁。因“刺激性干咳1月”于2019年1月1日就诊于青岛大学附属医院。2019年1月24日外院胸部计算机断层扫描（computed tomography, CT）提示左肺下叶占位（55 mm×58 mm），考虑肺癌伴癌性淋巴管炎；纵隔、左肺门多发轻度肿大淋巴结；双侧肺气肿、肺大泡。2019年1月29日外院支气管镜活检，病理：低分化鳞癌。基因检测：表皮生长因子受体（epidermal growth factor receptor, *EGFR*）（-）、原癌基因1酪氨酸激酶（*C-ros* oncogene 1 receptor tyrosine kinase, ROS1）（-）、间变性淋巴瘤激酶（anaplastic lymphoma kinase, ALK）（-）、原癌基因丝氨酸/苏氨酸蛋白激酶（protooncogene serine/threonine-protein kinase, *Raf*）（-）。细胞程序性死亡受体1（programmed cell death 1, PD-1）免疫组化（22C3）阴性。头部增强磁共振成像（magnetic resonance imaging, MRI）、同位素全身骨扫描（emission computed tomography, ECT）和胸腹盆腔增强CT未见远处转移。患者既往吸烟指数50年*20支=1, 000。冠心病10余年，2型糖尿病10余年，双侧颈动脉狭窄4余年。诊断：左肺低分化鳞癌T3N3M0 Ⅲc期，纵隔淋巴结转移EGFR/ALK/ROS1/RAF阴性，PD-L1（22C3）阴性，体力状况（performance status, PS）评分1分。

### 1.2 免疫检查点抑制剂相关肺炎（checkpoint inhibitor pneumonitis, CIP）前抗肿瘤治疗过程

2019年2月-2019年5月于青岛大学附属医院给予4个周期化疗联合抗血管生成治疗（重组人血管内皮抑制素注射液30 mg d1-d7+脂质体紫杉醇270 mg d1+顺铂60 mg d1-d2），2个周期后疗效评价疾病稳定（stable disease, SD），4个周期后SD略增大，患者干咳明显减轻。2019年6月于广州医科大学复大医院行左下肺病灶冷冻消融术。之后从2019年6月-2019年9月于青岛同和医院接受免疫靶向治疗6个周期：纳武利尤单抗单药200 mg，14 d。6个周期后疗效评价SD。2019年9月15日第7周期免疫治疗后患者出现轻度活动后憋气，无发热，未重视。2019年9月30日第8周期免疫治疗后患者憋气进行性加重伴干咳加重，出现低热，体温37.5 ℃左右。2019年10月5日青岛大学附属医院胸部CT：双肺感染性病变可能性大。先后接受头孢类抗生素口服和左氧氟沙星静脉给药抗感染治疗无效。2019年10月22日患者接受第9周期免疫治疗后憋气进一步加重，床上翻身可导致憋气。2019年10月28日于青岛市中心医院胸部CT：双肺间质性改变。当日急诊动脉血气分析：氧分压72 mmHg，二氧化碳分压27 mmHg。结合临床症状诊断为急性间质性肺炎，1型呼吸衰竭。收入青岛市中心医院呼吸科重症加强护理病房（intensive care unit, ICU）治疗。图 1可见CIP前后肺部影像学变化。

**图 1:**
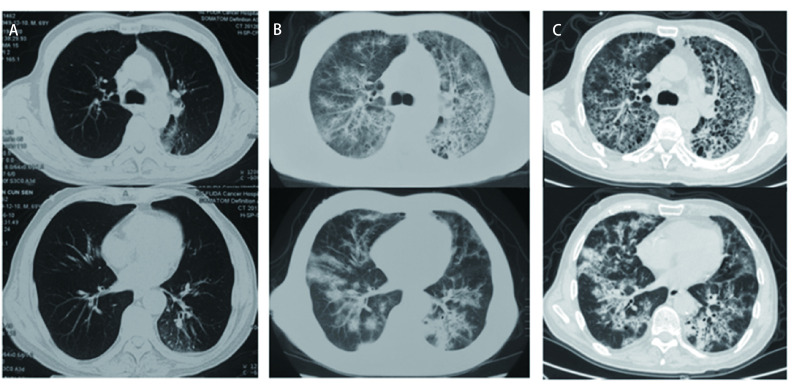
CIP发病前后肺部CT。A：第6周期纳武利尤单抗治疗后胸部CT（2019年6月10日）：表现为肺气肿、肺大泡和局限性淋巴管炎；B：第8周期纳武利尤单抗治疗后胸部CT（2019年10月5日）：表现为弥漫的磨玻璃样影伴网格状改变以及沿支气管分布的结节状斑片状实变影；C：第9周期纳武利尤单抗治疗后胸部CT（2019年10月28日）：表现为弥漫的磨玻璃样影伴网格状改变以及沿支气管分布的结节状斑片状实变影。 Pulmonary CT before and after the onset of CIP. A: Chest CT after treatment with Nivolumab in cycle 6 (June 10, 2019): emphysema, pulmonary bullae and localized lymphangitis; B: Chest CT after treatment with Nivolumab in cycle 8 (October 5, 2019): diffuse ground glass opacity with grid like changes and nodular patchy consolidation along the bronchus; C: Chest CT after treatment with Nivolumab in cycle 9 (October 28, 2019): diffuse ground glass opacity with grid like changes and nodular patchy consolidation along the bronchus. CIP: immune checkpoint inhibitor associated pneumonia; CT: computed tomography.

### 1.3 CIP治疗过程

入院查体：体温37.6 ℃，脉搏110次/min，呼吸30次/min，血压139 mmHg/75 mmHg，PS评分4分。憋喘貌，皮肤黏膜无紫绀，颈静脉无怒张，胸壁无皮下气肿，双肺叩诊呈清音，肺下界活动度正常，双肺呼吸音低，可闻及干湿性啰音，未闻及胸膜摩擦音。入院查血常规见白细胞及中性粒细胞升高：白细胞（white blood cell, WBC）计数16.07×10^9^/L，中性粒细胞比值80.9%，C反应蛋白（C-reactive protein, CRP）升高为108.54 mg/L。T淋巴细胞分类：CD4^+^ T细胞百分比升高46.3%，CD8^+^ T细胞降低13.7%。治疗方案：高流量吸氧+甲强龙冲击治疗+抗感染治疗。第1周注射用甲泼尼龙琥珀酸钠80 mg静脉给药*bid*（入院体重68 kg，2.35 mg/kg/d），1周后改为甲基强的松龙琥珀酸钠32 mg口服*bid*，并逐渐减量至6周后停药。由于初始不能排除感染性疾病以及甲强龙冲击造成的免疫抑制，给予莫西沙星抗细菌，卡泊芬净抗真菌，奥司他韦+更昔洛韦抗病毒治疗，1周后排除感染后停用药抗感染药物。患者进行了13种呼吸道细菌核酸检测、降钙素原、血培养和痰培养均为阴性，排除了细菌感染。巨细胞病毒DNA，呼吸道病原IgM抗体8项检测，患者乙型流感病毒IgM为弱阳性，不能完全排除病毒感染，因此给予了奥司他韦和更昔洛韦联合抗病毒治疗。真菌D-葡聚糖检测和曲霉菌免疫学实验、痰液真菌培养均不支持真菌感染。痰查抗酸杆菌及结核杆菌DNA检测均不支持结核感染。疟原虫免疫学检测排除疟疾。呼吸道病原体核酸检测和病原体IgM均不支持支原体和衣原体的感染。ENA抗体谱15项+抗核抗体检测不支持自身免疫性疾病造成的肺间质改变。心电图结合肺循环动力学检测均不支持肺栓塞，但由于患者体力状态不能耐受CT下肺动脉造影检查和肺通气灌注扫描检查，因此不能完全排除肺栓塞，故给予了抗凝治疗1周。期间自身抗体ANA谱（16项）均为阴性，排除自身免疫性肺炎。确诊为免疫检查点抑制剂相关肺炎3级。治疗1周后患者憋气好转，经皮氧分压由2019年10月28日61 mmHg改善为2019年11月4日94 mmHg，肺部CT炎症表现减轻。于2019年11月5日出院。呼吸科ICU出院后停用免疫靶向治疗，患者继续低流量吸氧并口服甲强龙片逐渐减量。2019年12月1日起口服吡非尼酮200 mg *tid*，1周后加量至300 mg *tid*，患者出现1级恶心，故继续300 mg *tid*的剂量维持治疗至今。患者憋气症状逐渐改善，2020年2月起停用低流量吸氧。2020年6月起PS评分1分，肺部间质性改变明显好转。图 2可见CIP治疗前后胸部CT影像学变化过程。

**图 2:**
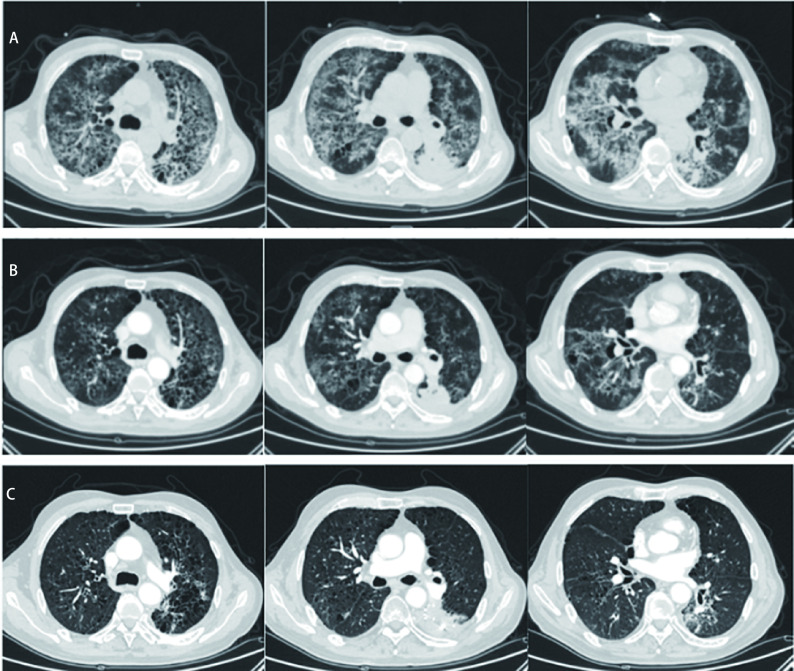
CIP治疗前后胸部CT。A：治疗前胸部CT（2019年10月28日）；B：糖皮质激素大剂量冲击治疗后胸部CT（2019年11月18日）；C：吡非尼酮治疗8月余后胸部CT（2020年7月14日）； Chest CT before and after treatment of CIP. A: Chest CT before treatment (October 28, 2019); B: Chest CT after high-dose glucocorticoid pulse therapy (November 18, 2019); C: Chest CT after pirfenidone treatment for more than 8 months (July 14, 2020).

### 1.4 确诊CIP后的抗肿瘤治疗过程

2019年11月-2020年1月青岛市中心医院给予白蛋白结合型紫杉醇单药化疗（200 mg d1, d8），2个周期及4个周期后疗效评价SD，患者过程中出现口腔黏膜炎3级，指尖麻木1级。2020年1月-2020年3月患者因疫情原因拒绝回医院化疗。2020年3月9日青岛市中心医院CT：左肺下叶占位明显增大，疗效评价疾病进展（progressive disease, PD）。患者拒绝化疗。2020年3月至今，安罗替尼8 mg口服*qd*，患者服药后出现1级乏力。2020年5月14日复查胸部CT疗效评价SD略增大。2020年5月25日行左肺癌动脉栓塞术，2020年5月29日行左肺癌碘125粒子植入术。2020年7月14日胸部CT疗效评价部分缓解（partial response, PR）。2020年9月22日复查胸部CT疗效评价PD，左下肺出现新发病灶，纵隔淋巴结较前增大。图 3可见近期治疗的CT评价情况。图 4为时间线。

**图 3:**
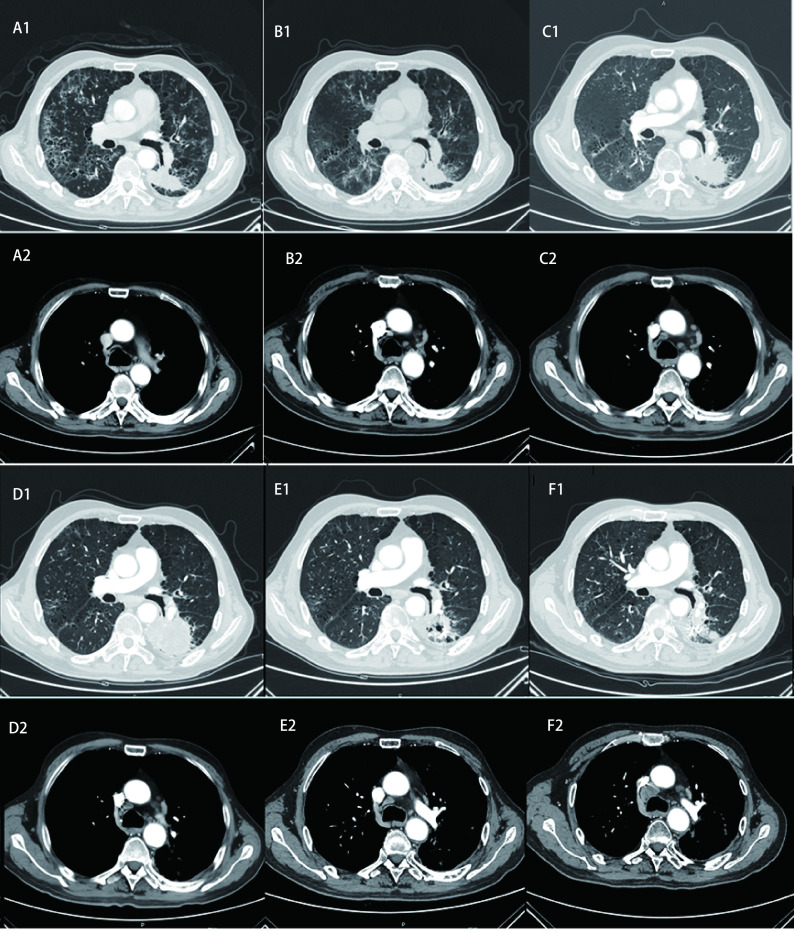
确诊CIP后抗肿瘤治疗CT疗效评价。A：白蛋白结合型紫杉醇化疗前基线CT（2019年11月18日）；B：白蛋白结合型紫杉醇化疗4个周期后CT（2020年1月16日），疗效评价SD；C：因疫情中断化疗2月后复查CT（2020年3月9日），肺部病灶进展；D：安罗替尼2个周期后CT（2020年5月14日），疗效评价SD（左下肺病灶略增大）；E：安罗替尼+左下肺病灶动脉栓塞术+碘125粒子植入术后CT（2020年7月14日），疗效评价PR；F：继续安罗替尼2个周期后CT（2020年9月22日），疗效评价PD（左下肺新发病灶，纵隔淋巴结增大）。 CT evaluation of anti-tumor therapy after diagnosis of CIP. A: Baseline CT before albumin binding paclitaxel chemotherapy (November 18, 2019); B: CT after 4 cycles of albumin binding paclitaxel chemotherapy (January 16, 2020). Efficacy evaluation was SD; C: CT scan after 2 months interruption of chemotherapy due to COVID-19 pandemic situation (March 9, 2020). The left lower lung lesion progressed; D: CT after 2 cycles of Anlotinib (May 14, 2020). Efficacy evaluation was SD (slightly enlarged left lower lung lesion); E: CT after Anlotinib+arterial embolization+iodine 125 seed implantation of left lower pulmonary lesion (July 14, 2020). Efficacy evaluation was PR; F: CT after sequential 2 cycles of Anlotinib (September 22, 2020). Efficacy evaluation was PD (newlesion in left lower lung, mediastinal lymph node enlargement). SD: stable disease; PD: progressive disease.

**图 4:**
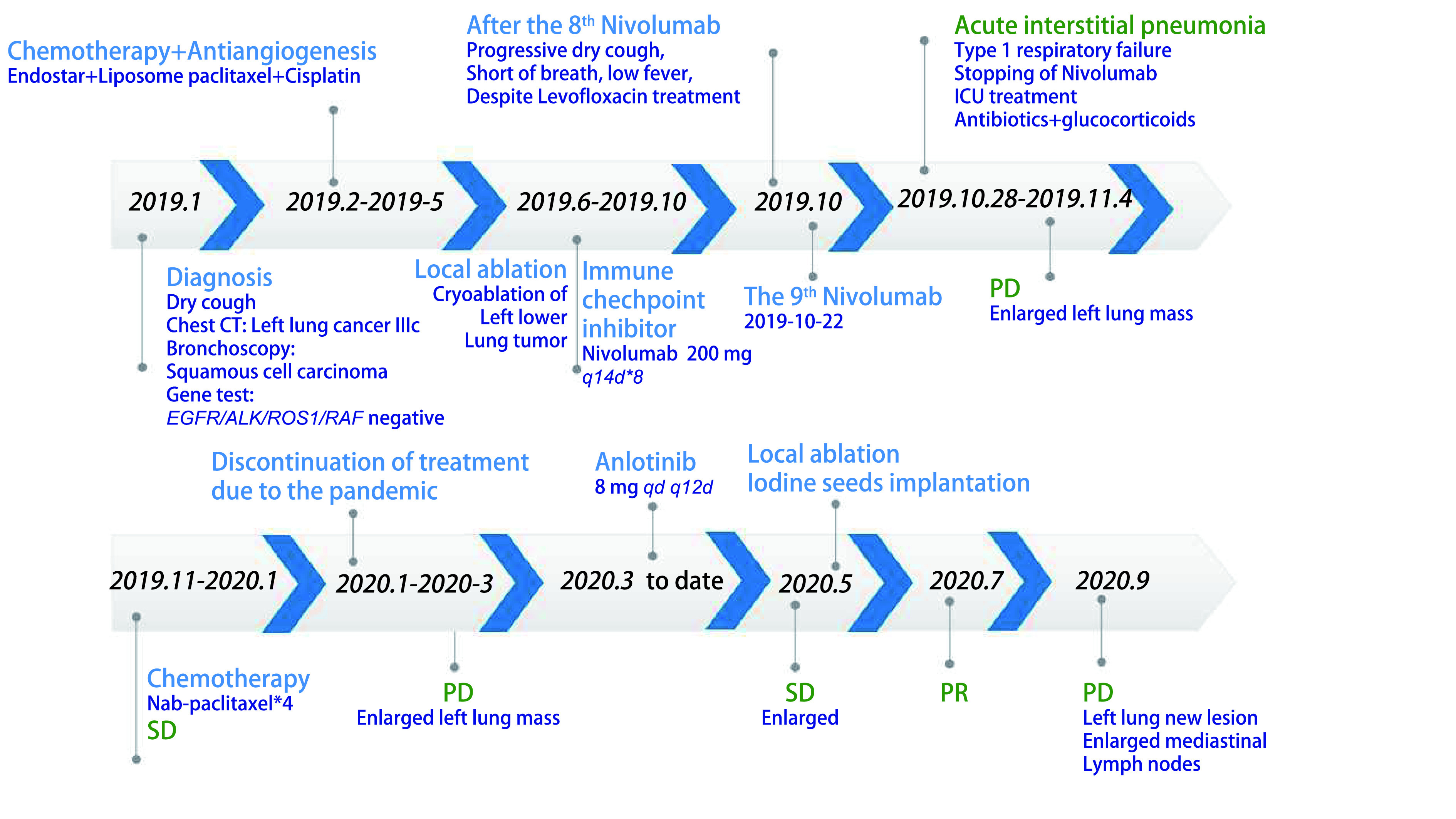
患者治疗过程时间线 Time line of treatment. PR: partial response.

## 2 讨论

CIP是由免疫检查点抑制剂（immune checkpoint inhibitor, ICI）引起的免疫相关的肺损伤，是免疫治疗相关死亡的独立危险因素，应当引起临床的高度重视^[1, 2]^。

患者在免疫靶向治疗12周（第6周期后）肺部CT未见肺间质改变，该患者在出现明确的间质性肺炎症状前2周已经出现活动后憋气等运动耐量减低的表现，但未重视，导致病情进一步恶化。免疫治疗开始后4个月（第8周期后）肺部CT呈现间质性肺炎的表现，伴发热、憋气等典型症状，符合间质性肺炎的诊断。按照《免疫检查点抑制剂相关肺炎诊治专家共识》^[1]^，要做出免疫检查点抑制剂相关肺炎的诊断，需要同时符合以下三个条件：①免疫检查点抑制剂用药史，患者符合；②间质性肺炎的影像学表现，患者符合；③鉴别诊断排除以下情况：肺部感染、肺部肿瘤进展、其他原因引起的肺间质性疾病、肺血管炎、肺栓塞、肺水肿等。初始对患者进行多种病原学及免疫学检测，除乙型流感病毒IgM（±）以外均为阴性。相关指标初步排除自身免疫性疾病。由于不能完全排除肺栓塞，给予了抗凝治疗1周。按照专家共识，明确诊断为“免疫检查点抑制剂相关肺炎3级”，初期治疗给予了糖皮质激素大剂量冲击治疗改口服后缓慢减量至停药。按照专家共识^[1, 2]^在感染无法完全排除的CIP的治疗起始阶段在激素同时进行了抗细菌、真菌和病毒的治疗。患者在以上治疗后影像学和临床症状均有部分改善。

CIP发病初期使用糖皮质激素可显著减轻肺部炎症渗出，但糖皮质激素在疾病后期肺部发生纤维化阶段作用甚微。如何治疗CIP患者急性期之后的肺纤维化来进一步改善肺功能？这是临床实践中亟待解决的问题。本病例除按照指南进行治疗之外，在糖皮质激素减量阶段开始并持续至今，接受了长达11个月的吡非尼酮的抗肺间质纤维化治疗，期间肺部影像学和临床症状持续改善，提示吡非尼酮治疗CIP有效，为CIP治疗提出了新的治疗方向。

吡非尼酮（Pirfenidone）在多项研究中被证实可治疗特发性肺纤维化（idiopathic pulmonary fibrosis, IPF），延缓肺功能恶化，甚至改善肺功能，且具有良好的耐受性和安全性^[3-5]^。《中国结缔组织病相关间质性肺病诊断和治疗专家共识》推荐吡非尼酮用于IPF的治疗^[6]^。在14例合并IPF的非小细胞肺癌患者的回顾性研究中，吡非尼酮与一线化疗联合用药，或在后线治疗中联合免疫检查点抑制剂均表现出良好的安全性，14例患者在治疗过程中均未出现IPF的加重^[7]^。IPF和CIP的发病机制有共通之处，转化生长因子β（transforming growth factor‐β, TGF‐β）在IPF和CIP的发病机制中均发挥核心作用^[8, 10]^。吡非尼酮的机制是抑制TGF‐β的产生，从而抑制肺间质纤维化^[8]^。吡非尼酮还能够通过抑制非小细胞肺癌的上皮间质转化（epithelial‐mesenchymal transition, EMT）发挥抗肿瘤的作用^[9]^。因此本病例中长期口服吡非尼酮耐受性好，而且获得肺部影像学及临床症状的改善。

本病例初始PD-1单抗治疗有效，经积极治疗后CIP症状已经得到明显缓解。目前肿瘤进展，患者拒绝化疗，缺乏更多的治疗选择。因此，下一步治疗方案为：PD-L1单抗联合吡非尼酮。依据如下：

按照专家共识，既往ICI治疗后出现≥3级免疫相关不良反应（immune related adverse effect, irAE）的患者应考虑永久停用ICIs^[1, 2]^。但临床实践中仍然有很多患者在irAE后再次使用ICIs，ICIs再挑战后的所有级别irAE的发生率在50%左右，3级-4级irAE的发生率在18%-35%左右，所有患者的irAE在及时治疗后均可得到明显缓解，总体安全性可控^[11-13]^。再挑战距离首次ICI治疗时间长的再次发生irAE的可能性较低^[13]^。在ICIs再挑战的2个非小细胞肺癌小样本研究中，再挑战的客观缓解率为5.9%-13%，疾病控制率可达到58.8%^[12, 14]^。有两个小样本研究对既往ICI疗效与再挑战疗效的相关性进行了分析，发现既往ICI疗效SD的患者疾病控制率可达到77.8%，甚至优于既往ICI疗效为PR的患者^[12, 14]^。本病例患者目前距离初次ICI治疗已接近1年时间，既往ICI疗效为SD，因此评估该患者ICIs再挑战治疗的安全性可控，有获益可能。较多的临床数据表明，PD-L1单抗治疗的肺炎发生率低于PD-1单抗^[1, 2, 15]^，因此计划在本病例的ICI再挑战中将PD-1单抗转换为PD-L1。

本病例报道的意义在于，该患者出现CIP之后长期口服吡非尼酮治疗肺纤维化，耐受性和安全性好，且取得较好的疗效，为CIP治疗提出了新的治疗方向，本病例报道为目前吡非尼酮治疗CIP的第1例报道。局限性在于，该病例仅为个案，说服力不足，仍需更多的临床实践和严格设计的临床研究来证实吡非尼酮治疗CIP的疗效。
